# Relevance of methylenetetrahydrofolate reductase gene variants C677T and A1298C with response to fluoropyrimidine-based chemotherapy in colorectal cancer: a systematic review and meta-analysis

**DOI:** 10.18632/oncotarget.24933

**Published:** 2018-07-27

**Authors:** Lei Zhong, Xia He, Yuan Zhang, Jun-Lan Chuan, Min Chen, Shao-Min Zhu, Qian Peng

**Affiliations:** ^1^ Personalized Drug Therapy Key Laboratory of Sichuan Province, Hospital of the University of Electronic Science and Technology of China and Sichuan Provincial People's Hospital, Sichuan 610072, China; ^2^ Department of Anesthesiology, East Ward, Sichuan Academy of Medical Sciences and Sichuan Provincial People's Hospital, Sichuan 610072, China; ^3^ Cancer Center, Sichuan Academy of Medical Sciences and Sichuan Provincial People's Hospital, Sichuan 610072, China

**Keywords:** MTHFR, fluoropyrimidine, chemotherapy response, meta-analysis, polymorphism

## Abstract

Methylenetetrahydrofolate reductase (MTHFR) is a critical enzyme influencing the metabolism of fluoropyrimidines. The relevance of *MTHFR* polymorphisms with the clinical response to fluoropyrimidine-based chemotherapy has been explored, but the results remain controversial. Thus, a meta-analysis was performed to provide a comprehensive estimate in this account. Relevant studies were identified through PubMed, Embase and Web of Science databases from inception up to May 2017. Odds ratios (ORs) with corresponding 95% confidence intervals (CIs) were applied to assess the strength of association. A total of 2118 colorectal cancer patients from 21 studies were included in the meta-analysis. Overall, there was no significant association between *MTHFR* C677T (rs1801133) or A1298C (rs1801131) polymorphisms and the clinical response to fluoropyrimidine-based chemotherapy under all of the three genetic models (allele model, dominant model, and recessive model) and stratification analysis, except for the retrospective study subgroup in the dominant model of *MTHFR* C677T and the “5-Fu *+* FA” treatment group in the allele contrast of *MTHFR* A1298C. No or moderate heterogeneity was observed in all genetic models. This meta-analysis suggested that *MTHFR* polymorphisms could not be considered as reliable factors for predicting the clinical response to fluoropyrimidine-based chemotherapy in colorectal cancer patients.

## INTRODUCTION

Fluoropyrimidines, such as 5-fluorouracil (5-FU), and the oral prodrugs capecitabine and tegafur have been widely used in the treatment of a variety of solid cancers for a long time, especially colorectal cancer (CRC) [1–3]. Fluoropyrimidine drugs themselves have no anti-tumor activity, but they can be metabolized into fluorodeoxyuridine monophosphate (FdUMP). FdUMP could further form the ternary complex with 5, 10-methylenetetrahydrofolate (5, 10-MTHF) and thymidylate synthase (TS), thereby inhibiting the activity of TS. This prevents the conversion of 2′-deoxyuridine-5′-monophosphate into 2′- deoxythymidine-5′-monophosphate, the latter of which is an essential precursor for DNA synthesis [4]. There are many factors influencing the metabolism of fluoropyrimidines, among them, the polymorphism of metabolism-related genes of fluoropyrimidine is one of the most pivotal factors.

Methylenetetrahydrofolate reductase (MTHFR), the most critical enzyme in folate-metabolizing pathway, catalyzes the irreversible conversion of 5, 10- methylenetetrahydrofolate (5, 10-MTHF) to 5-methyltetrahydrofolate, and reduces the amount of 5, 10-MTHF available for binding to FdUMP and TS [5]. Therefore, MTHFR plays a key role in the catabolism of fluoropyrimidines to the active metabolites. The activity of MTHFR may be an important factor for predicting the clinical response to fluoropyrimidine-based chemotherapy. The coding gene *MTHFR* locates in chromosome 1p36.3, and is highly polymorphic [6]. Two common functional polymorphisms in the *MTHFR* gene, C677T (rs1801133, A222V) and A1298C (rs1801131, E429A), have been identified the main variants affecting the activity of this enzyme [7, 8]. Thus, *MTHFR* C677T and A1298C polymorphisms are potential predictors for the clinical response to fluoropyrimidine-based chemotherapy.

Although theoretically *MTHFR* gene polymorphisms are closely related to the efficacy of fluoropyrimidines, in fact the available evidence from the genetic association studies in clinic was weak and the published results were inconsistent. This discordance also existed in the existing meta-analysis. The systematic review conducted by Elias Zintzaras and colleagues indicated that *MTHFR* C677T and A1298C gene polymorphisms could not be considered as reliable predictors of response to fluorouracil-based chemotherapy in patients with colorectal cancer [9]. However, another meta-analysis in colorectal and esophageal cancer, as well as a systematic review in gastric cancer, showed the opposite result [10, 11]. In this account, an update systematic review and meta-analysis containing 11 novel studies was carried out to further comprehensively estimate the correlation of MTHFR polymorphisms with the clinical response to fluoropyrimidine-based chemotherapy in CRC patients.

## RESULTS

### Study characteristics

**Figure 1:**
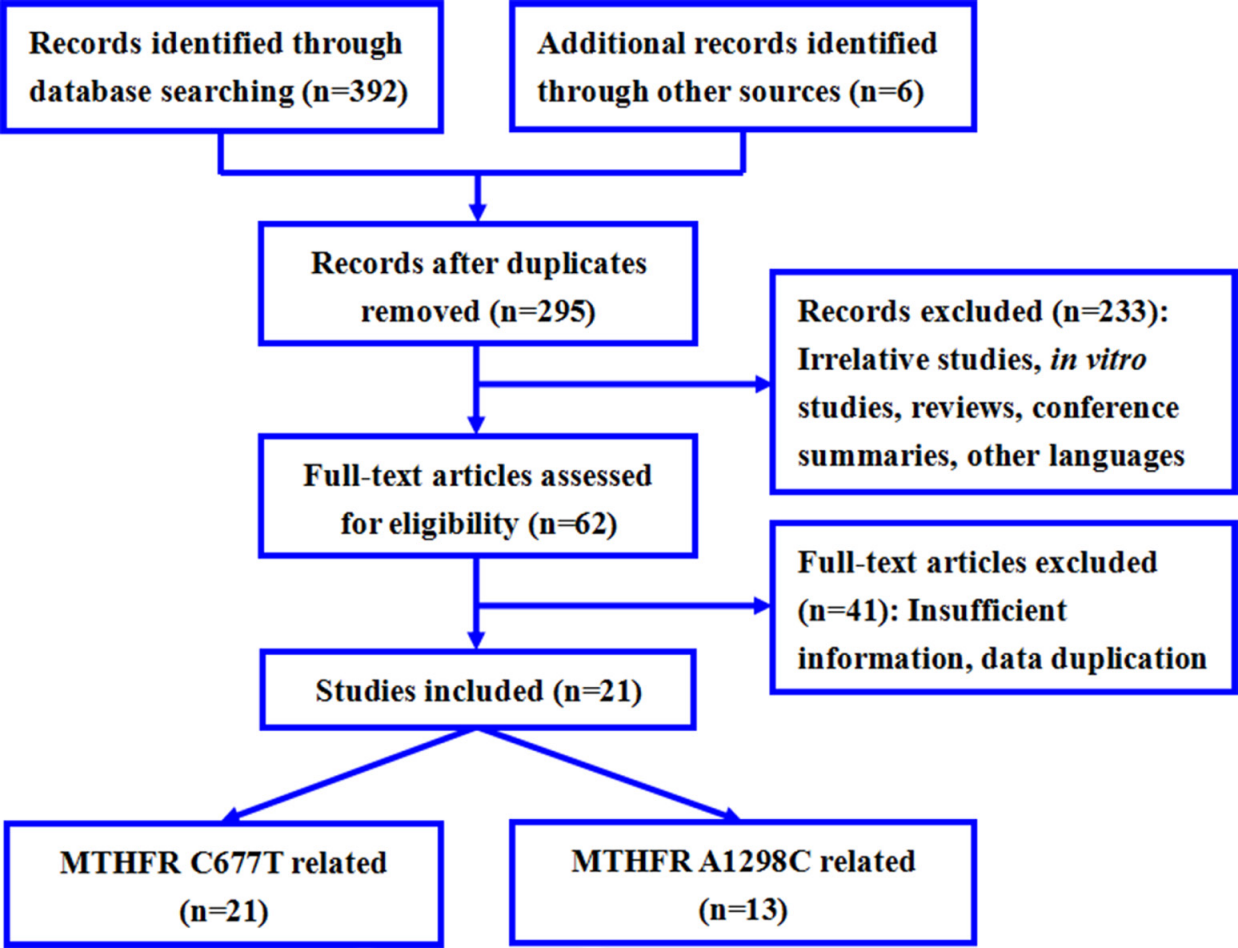
Flow diagram of study selection

This study is based on meta-analysis of observational studies in epidemiology (MOOSE). The flowchart of study selection was shown in Figure 1. A total of 295 potential relevant publications were retrieved from the databases. According to the inclusion/exclusion criteria, data from 21 studies that investigated the association between *MTHFR* C677T or A1298C polymorphisms and response to fluoropyrimidine-based chemotherapy in CRC patients were collected for the meta-analysis [12–32]. The characteristics of 21 eligible studies were shown in Table 1. Studies were published between 1999 and 2016, and sample sizes ranged from 43 to 238. Seven of twenty-one studies (33.3%) were conducted prospectively (Table 1). Of these publications, studies were conducted in three different ethnicities: Caucasian (fifteen studies), Asian (five studies), and mixed crowd (one study). All studies reported used fluoropyrimidines as treatment along with a combination of other interventions, such as folinic acid. Among the publications, 21 studies including 2118 patients reported tumor response events associated with *MTHFR* C677T polymorphism, and 13 studies provided 1496 patients for testing the association of *MTHFR* A1298C variant with response to chemotherapy (Table 1). In thirteen studies, responders were defined as patients with complete response (CR, disappearance of the disease), partial response (PR, decrease at least 50% in tumor load of the lesions) or stable disease (SD, without response or progression). In the remaining studies, responders were defined based on tumor regression grading (TRG), survival or early recurrence. Among them, the classification criteria of TRG was shown as follows: TRG1, absence of residual cancer and extensive fibrosis; TRG2, rare residual cancer cells scattered through the fibrosis; TRG3, increased residual cancer cells but fibrosis still predominating; TRG4, residual cancer outgrowing fibrosis; TRG5, absence of regressive changes. The quality of each eligible article was assessed by the Newcastle-Ottawa Scale (NOS), and all studies received a high NOS score (≥5, data not shown).

**Table 1:** Characteristics of the studies included in the meta-analysis

Study (year)	Ethnicity	Clinical data gathering	Patients, *n* (male%)	Age, mean (range)	Chemotherapy regimens	Definition of responders	Definition of non- responders	MTHFR SNP	Method of MTHFR SNP analysis	HWE reported and in equilibrium?	Ref.
Wisotzkey JD, *et al*. (1999)	Caucasian	Retrospective	51 (–)	–	5-Fu *+* folinic acid	Alive without any evidence of cancer	Dead or alive with cancer	C677T	PCR-RFLP	Not reported	12
Cohen V, *et al*. (2003)	Mixed crowd	Retrospective	43 (62.8)	59 (43–70)	5-Fu/LV, Capecitabine, UFT	CR, PR	SD, PD	C677T	PCR- Electrophoresis	Not reported	13
Etienne MC, *et al*. (2004)	Caucasian	Retrospective	98 (58.2)	64 (40–82)	5-Fu *+* folinic acid	CR, PR	SD, PD	C677T A1298C	PCR-HRM	Yes	14
Jakobsen A, *et al*. (2005)	Caucasian	Retrospective	88 (57)	62 (–)	5-Fu *+* leucovorin	CR, PR	SD, PD	C677T A1298C	PCR-RFLP	Not reported	15
Marcuello E, *et al*. (2006)	Caucasian	Prospective	94 (72)	68 (43–83)	5-Fu *+* Irinotecan, 5-Fu *+* leucovorin *+* oxaliplatin	CR, PR	SD, PD	C677T A1298C	RT-PCT	Not reported	16
Suh KW, *et al*. (2006)	Asian	Retrospective	54 (55.6)	57.8 (35–79)	FOLFOX	CR, PR, SD	PD	C677T	sequencing	Yes	17
Terrazzino S, *et al*. (2006)	Caucasian	Retrospective	125 (64)	60 (31–79)	5-FU, 5-Fu *+* leucovorin *+* oxaliplatin, 5-FU *+* CARBO	TRG 1-2	TRG 3-5	C677T A1298C	PCR- Electrophoresis	Yes	18
Capitain O, *et al*. (2008)	Caucasian	Retrospective	76 (60.5)	71 (39–88)	5-Fu *+* leucovorin	CR, PR	SD, PD	C677T A1298C	sequencing	Yes	19
Huang MY, *et al*. (2008)	Asian	Prospective	201 (58.7)	62 (33–75)	5-Fu *+* leucovorin	Non-early recurrence	Early recurrence	C677T	PCR-RFLP	Not reported	20
Ruzzo A, *et al*. (2008)	Caucasian	Prospective	146 (54.8)	61 (38–75)	FOLFIRI	CR, PR	SD, PD	C677T A1298C	PCR-RFLP	Yes	21
Balboa E, *et al*. (2010)	Caucasian	Prospective	65 (76.9)	64 (37–85)	5-Fu/Capecitabine	TRG 1-2	TRG 3-5	C677T A1298C	SnapShot	Yes	22
Etienne MC, *et al*. (2010)	Caucasian	Prospective	117 (55.6)	67 (31–80)	FOLFOX	CR, PR	SD, PD	C677T A1298C	PCR-HRM	Yes	23
Cecchin E, *et al*. (2011)	Caucasian	Retrospective	238 (66.8)	61 (20–79)	5-Fu, 5-Fu *+* platinum/ irinotecan/gefitinib	TRG 1-2	TRG 4-5	C677T A1298C	TaqMan assay	No	24
Lamas MJ, *et al*. (2011)	Caucasian	Retrospective	72 (69.4)	66.5 (32–80)	FOLFOX, FOLFOX *+* Cetuximab, FOLFOX *+* Bevacizumab	CR, PR, SD	PD	C677T A1298C	SnapShot	Yes	25
Hu-Lieskovan S, *et al*. (2011)	Caucasian	Retrospective	130 (57)	61 (33–83)	5-Fu *+* Cetuximab, Capecitabine *+* Cetuximab, Capecitabine *+* Oxaliplatin *+* Cetuximab	TRG 1	TRG 2-5	C677T A1298C	PCR-RFLP	Not reported	26
Budai B, *et al*. (2012)	Caucasian	Prospective	85 **(–)**	_	FOLFIRI *+* bevacizumab	CR, PR	SD, PD	C677T	PCR-RFLP	Yes	27
Chai HN, *et al*. (2012)	Asian	Prospective	73 (61.6)	59 (24–87)	FOLFOX	CR, PR	SD, PD	C677T	sequencing	Yes	28
Zhao J, *et al*. (2012)	Asian	Retrospective	154 (58.4)	56 (30–75)	FOLFOX, XELOX, FOLFIRI	CR, PR	SD, PD	C677T A1298C	sequencing	Not reported	29
Lamas MJ, *et al*. (2012)	Caucasian	Retrospective	93 (73.1)	67 (39–86)	5-Fu	TRG 1-2	TRG 3-5	C677T A1298C	SnapShot	Yes	30
Kumamoto K, *et al*. (2013)	Asian	Retrospective	63 (**65.1)**	65 (32–84)	FOLFOX	CR, PR	SD, PD	C677T	PCR-RFLP	Not reported	31
Boudaoud K, *et al*. (2016)	Caucasian	Retrospective	52 (59.6)	50.8 (23–70)	5-Fu *+* FA, Capecitabine	pCR *+* downstaging	_	C677T	PCR-RFLP	Not reported	32

*HWE*, Hardy-Weinberg equilibrium; *CR*, complete response; *PR*, partial response; *SD*, stable disease; *PD*, progressive disease; *pCR*, pathologic complete response; *TRG*, tumor regression grading; *PCR*, polymerase chain reaction; *RFLP*, restriction fragment length polymorphism; *HRM*, High Resolution Melting.

### Association of *MTHFR* C677T polymorphism with response to fluoropyrimidine-based chemotherapy

**Figure 2:**
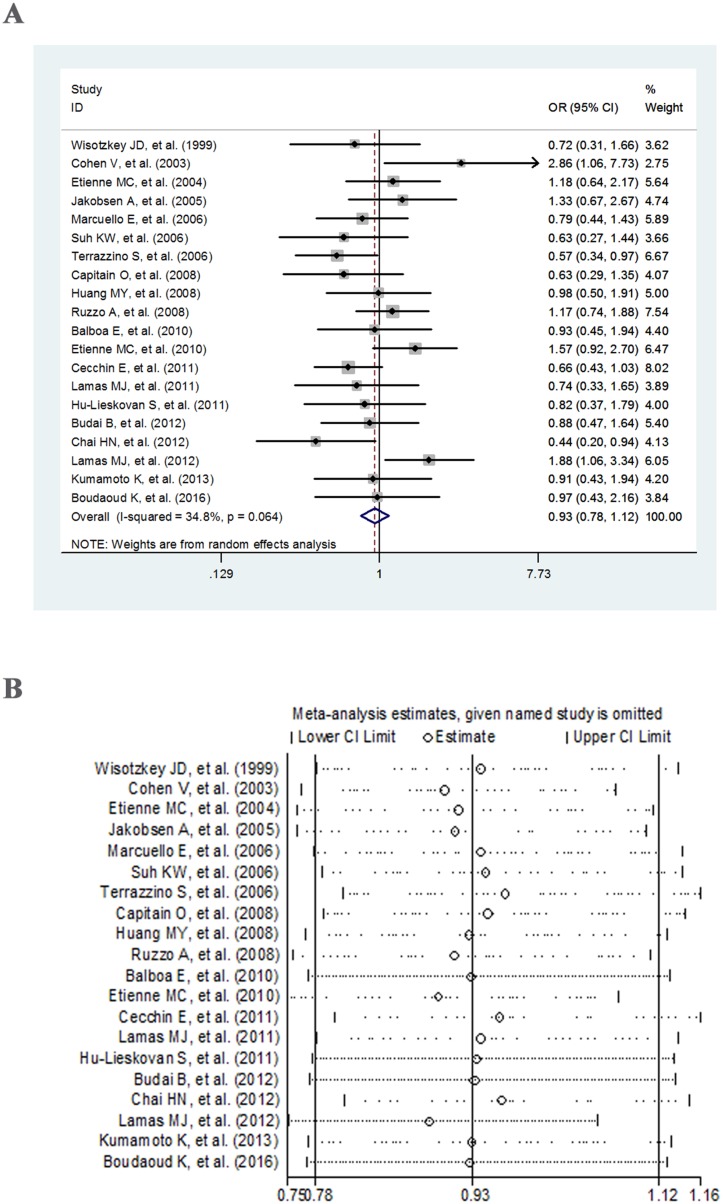
Forest plot (**A**) and sensitivity analysis (**B**) for the allele contrast of *MTHFR* C677T polymorphism and response to fluoropyrimidine-based chemotherapy.

The main results of meta-analysis and heterogeneity test for *MTHFR* C677T were summarized in Table 2. Overall, no significant association was found between *MTHFR* C677T polymorphism and response to fluoropyrimidine-based chemotherapy under all three genetic models: allele model (OR = 0.93, 95% CI = 0.78–1.12) (Figure 2A), dominant model (OR = 0.79, 95% CI = 0.63–1.00) (Supplementary Figure 1A), and recessive model (OR = 1.20, 95% CI = 0.91–1.57) (Supplementary Figure 2A). The Q-statistic and I^2^ index in the three models indicated moderate heterogeneity in allele and dominant models (25% < I^2^ < 50%), and no significant heterogeneity under recessive model (*P_Q_* = 0.151, I^2^ = 24.9%). When stratified by ethnicity, study type, clinical outcome and chemotherapy regimen, only the retrospective study subgroup in dominant model showed a significant association (OR = 0.69, 95% CI = 0.53–0.90). Moreover, the heterogeneity only reduced simultaneously when stratified by ethnicity under allele model (Table 2).

**Table 2:** Odds ratio with the corresponding 95% confidence interval, heterogeneity results, Egger’ test and Begg’ test for genetic contrasts of *MTHFR* C677T

Models	Population	No. studies	Random effects OR (95% CI)	*P*-value (*Q*-test)	I^2^ (%)	Egger’ test	Begg’ test
T versus C	All	20	0.93 (0.78–1.12)	0.064	34.8	0.766	0.721
	Caucasians	15	0.95 (0.78–1.15)	0.144	28.5	0.713	0.692
	Asians	4	0.73 (0.50–1.06)	0.404	0.0	0.325	0.308
	Prospective	7	0.96 (0.73–1.27)	0.212	28.4	0.102	0.230
	Retrospective	13	0.92 (0.72–1.18)	0.060	41.1	0.369	0.760
	ORR	12	0.98 (0.77–1.24)	0.114	34.6	0.978	0.373
	TRG	5	0.87 (0.57–1.35)	0.029	63.0	0.703	0.806
	5-Fu *+* FA	5	0.98 (0.71–1.34)	0.568	0.0	0.156	0.462
	FOLFOX	4	0.83 (0.46–1.50)	0.042	63.4	0.058	0.308
Dominant model	All	21	0.79 (0.63–1.00)	0.117	27.8	0.884	0.928
	Caucasians	15	0.79 (0.60–1.05)	0.108	32.5	0.408	0.692
	Asians	5	0.68 (0.46–1.02)	0.700	0.0	0.313	0.806
	Prospective	7	0.99 (0.67–1.47)	0.179	32.7	0.167	0.101
	Retrospective	14	0.69 (0.53–0.90)	0.306	13.4	0.273	0.189
	ORR	13	0.85 (0.62–1.15)	0.156	28.7	0.854	0.951
	TRG	5	0.65 (0.37–1.11)	0.071	53.6	0.757	1.000
	5-Fu *+* FA	5	0.75 (0.50–1.14)	0.733	0.0	0.622	0.806
	FOLFOX	4	0.78 (0.35–1.73)	0.056	60.4	0.164	0.734
Recessive model	All	20	1.20 (0.91–1.57)	0.151	24.9	0.389	0.315
	Caucasians	15	1.26 (0.88–1.82)	0.139	29.0	0.543	0.322
	Asians	4	0.67 (0.26–1.76)	0.464	0.0	0.519	0.308
	Prospective	7	0.92 (0.58–1.47)	0.641	0.0	0.481	0.368
	Retrospective	13	1.44 (0.89–2.33)	0.085	37.3	0.351	0.583
	ORR	12	1.22 (0.78–1.92)	0.195	25.3	0.320	0.537
	TRG	5	1.28 (0.63–2.61)	0.077	52.5	0.570	0.462
	5-Fu *+* FA	5	1.82 (0.73–4.51)	0.191	34.6	0.491	0.462
	FOLFOX	4	0.88 (0.35–2.25)	0.268	23.9	0.132	0.089

*ORR*, objective response rate (CR, PR, SD and PD) as end point; *TRG*, tumor regression grading as end point; *5-Fu + FA,* chemotherapy regimens including 5-Fu *+* folinic acid and 5-Fu *+* leucovorin; *FOLFOX*, fluorouracil *+* leucovorin *+* oxaliplatin.

### Association of *MTHFR* A1298C polymorphism with response to fluoropyrimidine-based chemotherapy

**Figure 3:**
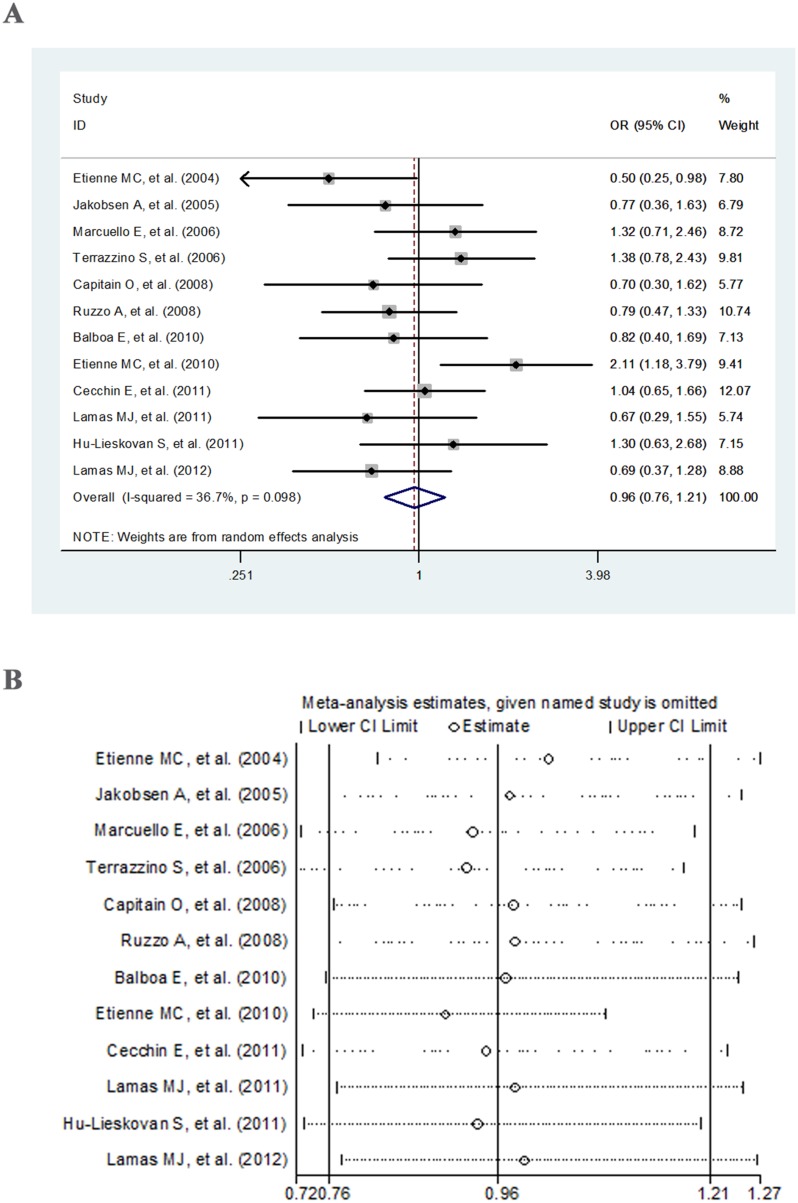
Forest plot (**A**) and sensitivity analysis (**B**) for the allele contrast of *MTHFR* A1298C polymorphism and response to fluoropyrimidine-based chemotherapy.

For the association between *MTHFR* A1298C polymorphism and response to fluoropyrimidine-based chemotherapy, the pooled results from meta-analysis showed no significant association in all of the three genetic models (Table 3, Figure 3A, and Supplementary Figures 3A and 4A). Moderate heterogeneity was observed in allele contrast and dominant model (25% < I^2^ < 50%; Table 3), but not in recessive model (*P_Q_* = 0.247, I^2^ = 20%; Table 3). In the subgroup analysis according to ethnicity, study type, clinical outcome and chemotherapy regimen, the association was still not significant except in the “5-Fu *+* FA” group of allele contrast (OR = 0.63, 95% CI = 0.41–0.97; Table 3). Moreover, the heterogeneity was evidently eliminated in the retrospective study group, TRG group and “5-Fu *+* FA” treatment group of allele and dominant models (*P_Q_* > 0.1, I^2^ < 25%; Table 3).

**Table 3:** Odds ratio with the corresponding 95% confidence interval, heterogeneity results, Egger’ test and Begg’ test for genetic contrasts of *MTHFR* A1298C

Models	Population	No. studies	Random effects OR (95% CI)	*P*-value (*Q*-test)	I^2^ (%)	Egger’ test	Begg’ test
C versus A	All	12	0.96 (0.76–1.21)	0.098	36.7	0.376	0.244
	Caucasians	12	0.96 (0.76–1.21)	0.098	36.7	0.376	0.244
	Asians	0					
	Prospective	4	1.17 (0.73–1.86)	0.067	58.0	0.997	1.000
	Retrospective	8	0.87 (0.68–1.12)	0.314	14.8	0.269	0.386
	ORR	7	0.90 (0.61–1.33)	0.033	56.2	0.394	0.548
	TRG	5	1.02 (0.79–1.33)	0.489	0.0	0.865	0.806
	5-Fu *+* FA	3	0.63 (0.41–0.97)	0.670	0.0	0.490	1.000
Dominant model	All	13	0.97 (0.73–1.28)	0.123	32.4	0.360	0.428
	Caucasians	12	0.98 (0.72–1.33)	0.093	37.3	0.335	0.451
	Asians	1					
	Prospective	4	1.20 (0.62–2.31)	0.041	63.7	0.965	0.734
	Retrospective	9	0.90 (0.68–1.19)	0.403	3.9	0.129	0.175
	ORR	8	0.86 (0.57–1.30)	0.070	46.5	0.555	0.711
	TRG	5	1.15 (0.81–1.64)	0.496	0.0	0.540	0.806
	5-Fu *+* FA	3	0.62 (0.37–1.07)	0.962	0.0	0.271	0.296
Recessive model	All	12	0.87 (0.58–1.30)	0.247	20.0	0.177	0.134
	Caucasians	12	0.87 (0.58–1.30)	0.247	20.0	0.177	0.134
	Asians	0					
	Prospective	4	1.40 (0.45–4.41)	0.146	44.3	0.168	0.734
	Retrospective	8	0.78 (0.44–1.38)	0.335	12.2	0.881	0.536
	ORR	7	1.05 (0.45–2.47)	0.197	30.3	0.438	0.368
	TRG	5	0.80 (0.42–1.51)	0.305	17.2	0.500	0.462
	5-Fu *+* FA	3	0.49 (0.13–1.86)	0.235	31.0	0.260	1.000

*ORR*, objective response rate (CR, PR, SD and PD) as end point; *TRG*, tumor regression grading as end point; *5-Fu + FA,* chemotherapy regimens including 5-Fu *+* folinic acid and 5-Fu *+* leucovorin.

### Sensitivity analysis and publication bias

The sensitivity of the overall results was assessed by sequential omission of individual studies. As indicated in Figure 2B, Figure 3B and Supplementary Figures 1–4B, there were no individual studies in all three models (allele model, dominant model and recessive model) that could significantly influence the combined results, indicating the reliability and stability of our results. In addition, we used Egger's test and Begg's test to assess the publication bias. As shown in Table 2 and Table 3, the *P* values were all greater than 0.05 in both tests under all of the three genetic models of *MTHFR* C677T or A1298C polymorphisms, suggesting no indication of significant publication bias.

**Figure 4:**
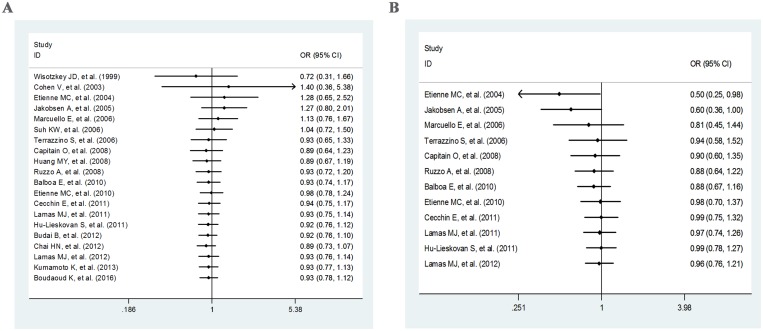
Cumulative meta-analysis for the allele contrast of *MTHFR* C677T (**A**) and A1298C (**B**) polymorphisms.

Cumulative meta-analysis in allele model of *MTHFR* C677T polymorphism exhibited that the OR increased from 0.72 in 1999 to 1.4 in 2003, and then reduced to 0.89 in 2008, followed by fluctuating around 1.0, but did not exceed 1.0 (Figure 4A). The association remained nonsignificant throughout the whole period. For the allele contrast of *MTHFR* A1298C polymorphism, the OR showed an upward trend overall (from 0.50 in 2004 to 0.96 in 2012), with fluctuation in the period of 2006–2012. The significant association just existed before 2005 (Figure 4B).

## DISCUSSION

The association of *MTHFR* polymorphisms with the clinical response to fluoropyrimidine-based chemotherapy in CRC patients is still controversial. Etienne-Grimaldi et al. has demonstrated that *MTHFR* genotypes linked to the clinical response to fluoropyrimidine-based treatment. Importantly, the score of favourable *MTHFR* alleles (677T and 1298C) was positively linked to response, with response rates of 37.1%, 53.3%, 62.5% and 80.0% in CRC patients bearing no, one, two or three favourable alleles, respectively [23]. While this correlation was not replicated in some other studies, which showed that no significant differences were identified between the polymorphisms of *MTHFR* and the efficacy of fluoropyrimidine-based treatment [30, 31]. Similarly, this discordance was also present in the *in vitro* studies [33, 34]. To quantitatively and comprehensively evaluate the effect of *MTHFR* C677T and A1298C polymorphisms on fluoropyrimidine-based chemotherapy in CRC patients, a meta-analysis including 21 studies was performed. The results of present meta-analysis suggested that there was no significant association between *MTHFR* C677T or A1298C polymorphisms and the clinical response to fluoropyrimidine-based chemotherapy in patients with CRC under allele, dominant and recessive models. Of note, when stratified by study type and chemotherapy regimen, the significant association could be observed in the retrospective study group of *MTHFR* C677T dominant model and the “5-Fu *+* FA” treatment group of *MTHFR* A1298C allele contrast. However, taking into account the authority of retrospective studies and the small number of studies included in the analysis, the results of these subgroup analysis require further assessed more scientifically.

The present meta-analysis has some limitations that need to be addressed. First, due to the lack of original data, our analysis was based on OR values without adjustment for other covariates such as age, gender, which may result in relatively low power to estimate the real association. This is also a general problem of meta-analysis when pooling data from primary studies. Second, some subgroup analysis, especially stratified by chemotherapy regimen, had insufficient statistical power to detect the association for the limited number of included studies. Finally, heterogeneity is a noticeable problem in this meta-analysis. Although it was moderate, potential sources of heterogeneity were not found absolutely by the sensitivity analysis and stratification analysis. When stratified by ethnicity, study type, clinical outcome and chemotherapy regimen, the heterogeneity just decreased simultaneously in both Caucasians and Asians subgroups under the allele model of *MTHFR* C677T (Table 2).

There are many factors contributing to the heterogeneity among studies except for ethnicity, study type, clinical outcome and chemotherapy regimen. Folate intake status is one of the most important influence factors [35, 36]. MTHFR is a critical enzyme in folate-metabolizing pathway, and folate status may affect the association of *MTHFR* polymorphisms with response to fluoropyrimidine-based treatment through gene-nutrition interaction. However, this effect was not assessed adequately in this study due to the unavailability of original data. The administration mode of fluoropyrimidines is also a factor influencing the efficacy of the agents. Fluoropyrimidines act in two different ways. Bolus fluoropyrimidines incorporate into RNA and preclude protein synthesis, while continuous infusion may have a preferential effect on TS [4]. The eligible studies in this meta-analysis used both modes of fluoropyrimidines administration. Additionally, fluoropyrimidines were combined with multiple chemotherapeutic agents in the studies included. Different combination regimens may cause the diversities in efficacy, thus contributing to the heterogeneity among studies.

In summary, the current meta-analysis found that *MTHFR* C677T and A1298C polymorphisms could not be considered as reliable factors for predicting the clinical response to fluoropyrimidine-based chemotherapy in patients with CRC. However, the results in present meta-analysis should be interpreted with cautiously due to the moderate heterogeneity in some genetic models. Therefore, well-designed prospective studies based on larger sample sizes are warranted to validate the present findings. Additionally, in view of the fact that fluoropyrimidines exert their effects through a multistep, multigenic cascade, hence, composite pharmacogenomics analysis may be more precise for efficacy prediction of fluoropyrimidine-based regimens.

## MATERIALS AND METHODS

### Search strategy

We searched PubMed, Embase and Web of Science databases from inception up to May 2017 using a combination of the following terms: “MTHFR” or “methylenetetrahydrofolate reductase”, “pharmacogenetic” or “polymorphism” or “genotype” or “variant” or “variation” or “mutant”, “fluorouracil” or “5-Fu” or “capecitabine” or “tegafur” or “fluoropyrimidine”, and “colon cancer” or “rectal cancer” or “colorectal cancer” or “CRC”. The search was restricted to articles in English-language. To identify more potentially relevant studies, a manual search for references cited in the eligible articles was also performed.

### Inclusion and exclusion criteria

The eligible studies in this meta-analysis fulfill the following inclusion criteria: (a) studies involving any type of colorectal cancer; (b) using chemotherapy regimens containing 5-Fu, capecitabine or tegafur; (c) using validated molecular methods for genotyping; (d) providing information on *MTHFR* polymorphism or estimated genetic effects on response to treatment. No restrictions were imposed on the design of the studies, which could have been prospective or retrospective studies. Studies investigating susceptibility, progression, or severity, and the case reports, letters, conference abstracts, meta-analysis, and reviews were excluded.

### Data extraction

Full reports of relevant studies were retrieved and independently extracted by two investigators (Yuan Zhang and Jun-Lan Chuan). The extracted data included first author's name, publication year, ethnicity of the study population, study design, distribution of gender and age in patients, clinical outcomes investigated, chemotherapy regimen, clinical response, genotype distribution of *MTHFR* and genotyping methods. Any discrepancies in data extraction were resolved by consensus.

### Assessment of study quality

The quality of the included studies was evaluated independently by two reviewers according to the Newcastle-Ottawa Scale (NOS) [37]. The NOS includes three parameters of quality for studies: selection of the study population, comparability of subjects, and exposure assessment, with scores ranging from 0 to 9. NOS scores of 0–4 and 5–9 were considered as low and high-quality studies, respectively.

### Statistical analysis

The strength of association between the *MTHFR* C677T or A1298C polymorphisms and clinical response was assessed by odds ratio (OR) and corresponding 95% confidence interval (CI) under the allele model (C677T: T vs. C; A1298C: C vs. A), dominant model (C677T: CT *+* TT vs. CC; A1298C: AC *+* CC vs. AA), and recessive model (C677T: TT vs. CC *+* CT; A1298C: CC vs. AA *+* AC). The heterogeneity between included studies was evaluated by the *Q*-test. *P* > 0.1 indicates that there is no significant heterogeneity. I^2^ statistic was also calculated to quantify the heterogeneity: I^2^ < 25 %, I^2^ = 25–50%, I^2^ = 50–75% and I^2^ > 75%, indicated no heterogeneity, moderate heterogeneity, large heterogeneity, and extreme heterogeneity, respectively. When *P_Q_* > 0.1 and I^2^ < 25%, the heterogeneity was considered to be nonsignificant and then the pooled OR and 95% CIs could be assessed by the fixed-effects model; otherwise, the random-effects model was used. Subgroup analyses were performed based on ethnicity (Caucasians and Asians), study type (prospective and retrospective), clinical outcome (objective response and TRG) and chemotherapy regimen, and only for groups reported in at least three independent studies. The sensitivity analysis was carried out by sequential omission of individual studies to assess the stability of the results. The publication bias was detected using Egger's regression test and Begg–Mazumdar adjusted rank correlation test. *P* < 0.05 indicated the presence of potential publication bias. Additionally, the cumulative meta-analysis was also carried out chronologically by publication year to observe the trend in estimated risk effect. All statistical analyses were conducted with the software STATA version 12.0 (Stata Corporation, College Station, TX, USA).

## SUPPLEMENTARY MATERIALS FIGURES


