# Supplementary material for: Relevance of methylenetetrahydrofolate reductase gene variants C677T and A1298C with response to fluoropyrimidine-based chemotherapy in colorectal cancer: a systematic review and meta-analysis
Source: Oncotarget. 2018 Jul 27;9(58):31291–301. doi: 10.18632/oncotarget.24933 (PMC6101282; doi:10.18632/oncotarget.24933)
Supplement: Supplementary file 1 [file oncotarget-09-31291-s001.pdf]

Relevance of methylenetetrahydrofolate reductase gene variants C677T and A1298C with response to fluoropyrimidine-based chemotherapy in colorectal cancer: a systematic review and meta-analysis

SUPPLEMENTARY MATERIALS

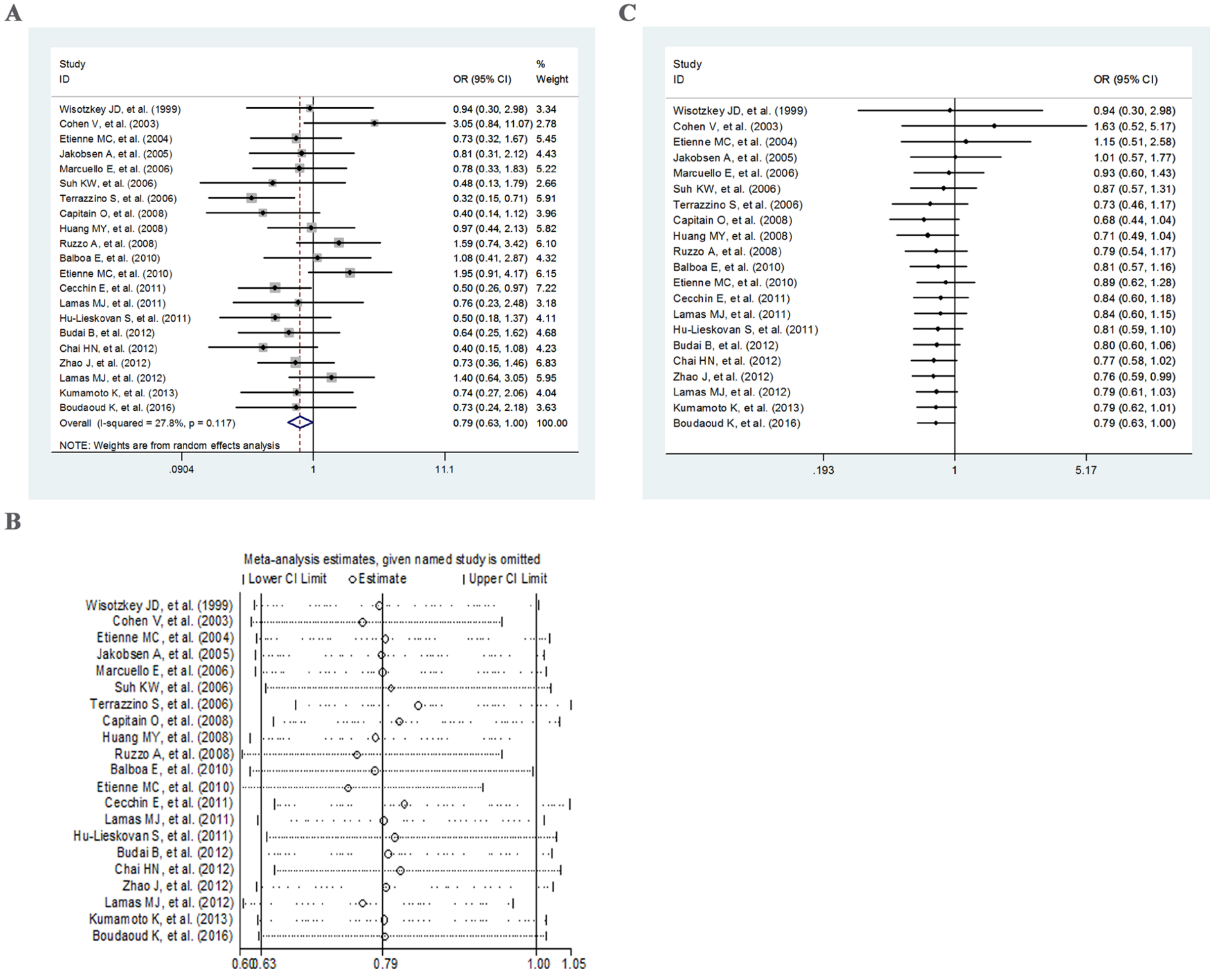

Supplementary Figure 1: Forest plot (**A**), sensitivity analysis (**B**) and cumulative meta-analysis (**C**) for the dominant model of *MTHFR* C677T polymorphism and response to fluoropyrimidine-based chemotherapy.

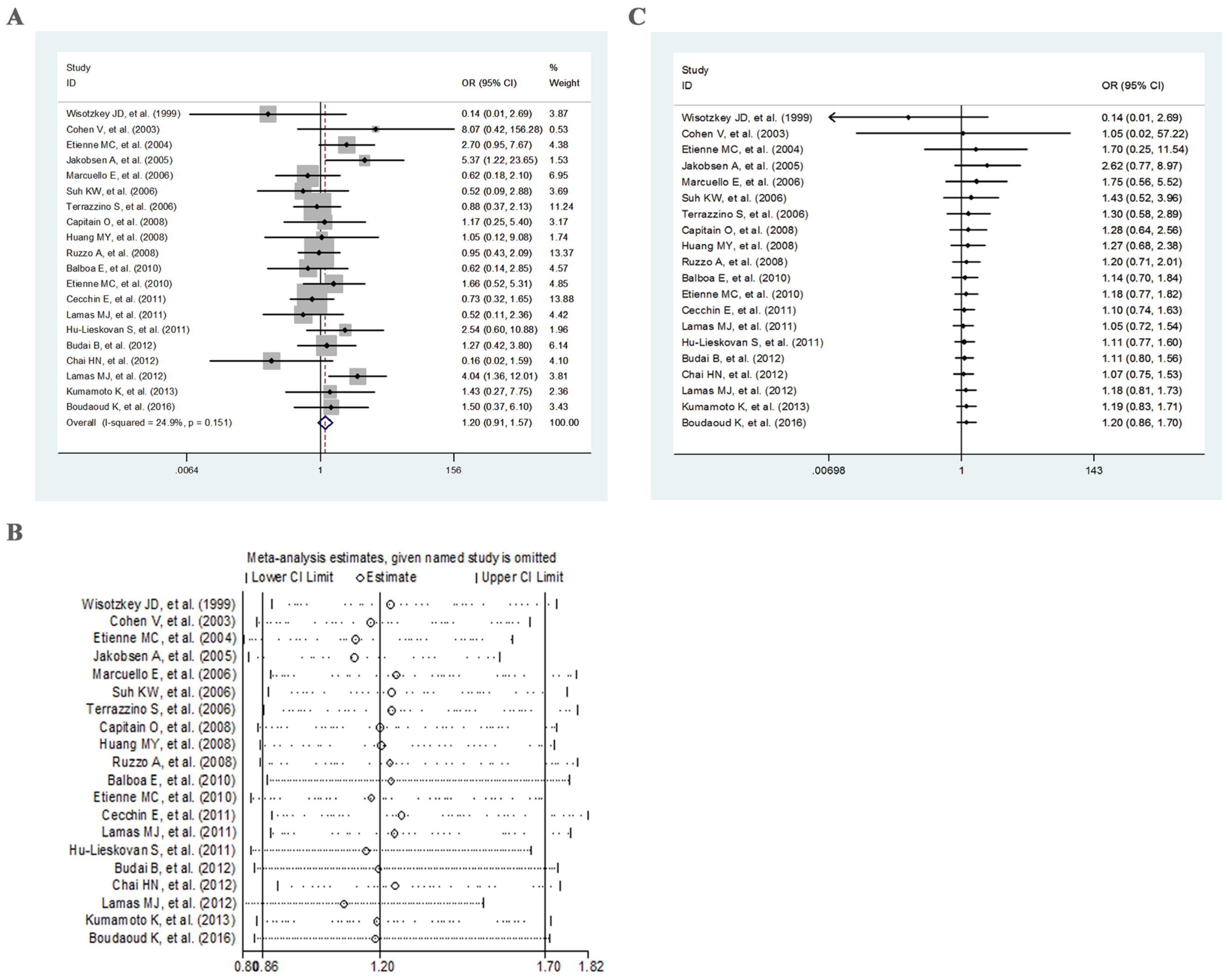

Supplementary Figure 2: Forest plot (**A**), sensitivity analysis (**B**) and cumulative meta-analysis (**C**) for the recessive model of *MTHFR* C677T polymorphism and response to fluoropyrimidine-based chemotherapy.

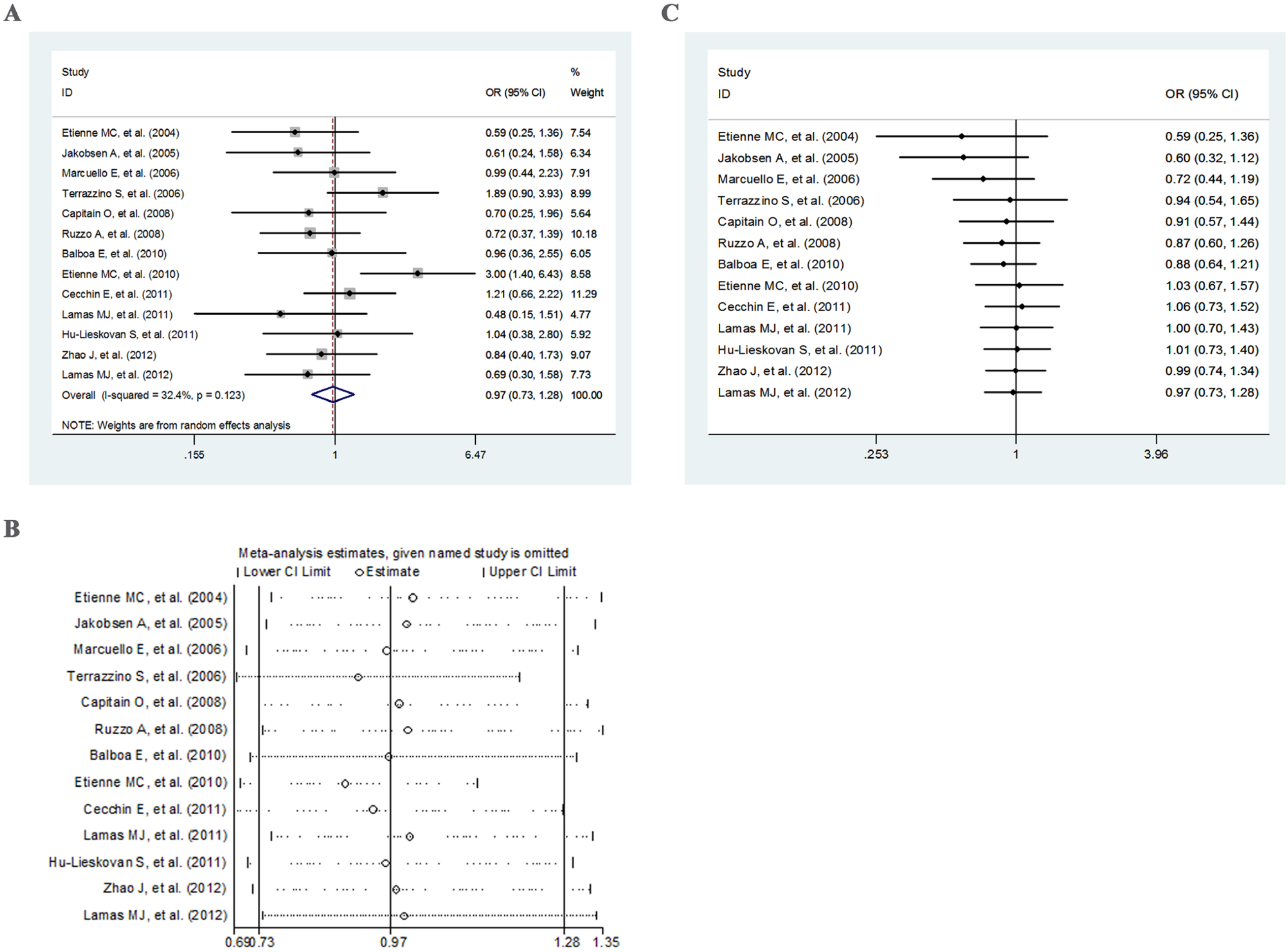

Supplementary Figure 3: Forest plot (**A**), sensitivity analysis (**B**) and cumulative meta-analysis (**C**) for the dominant model of *MTHFR* A1298C polymorphism and response to fluoropyrimidine-based chemotherapy.

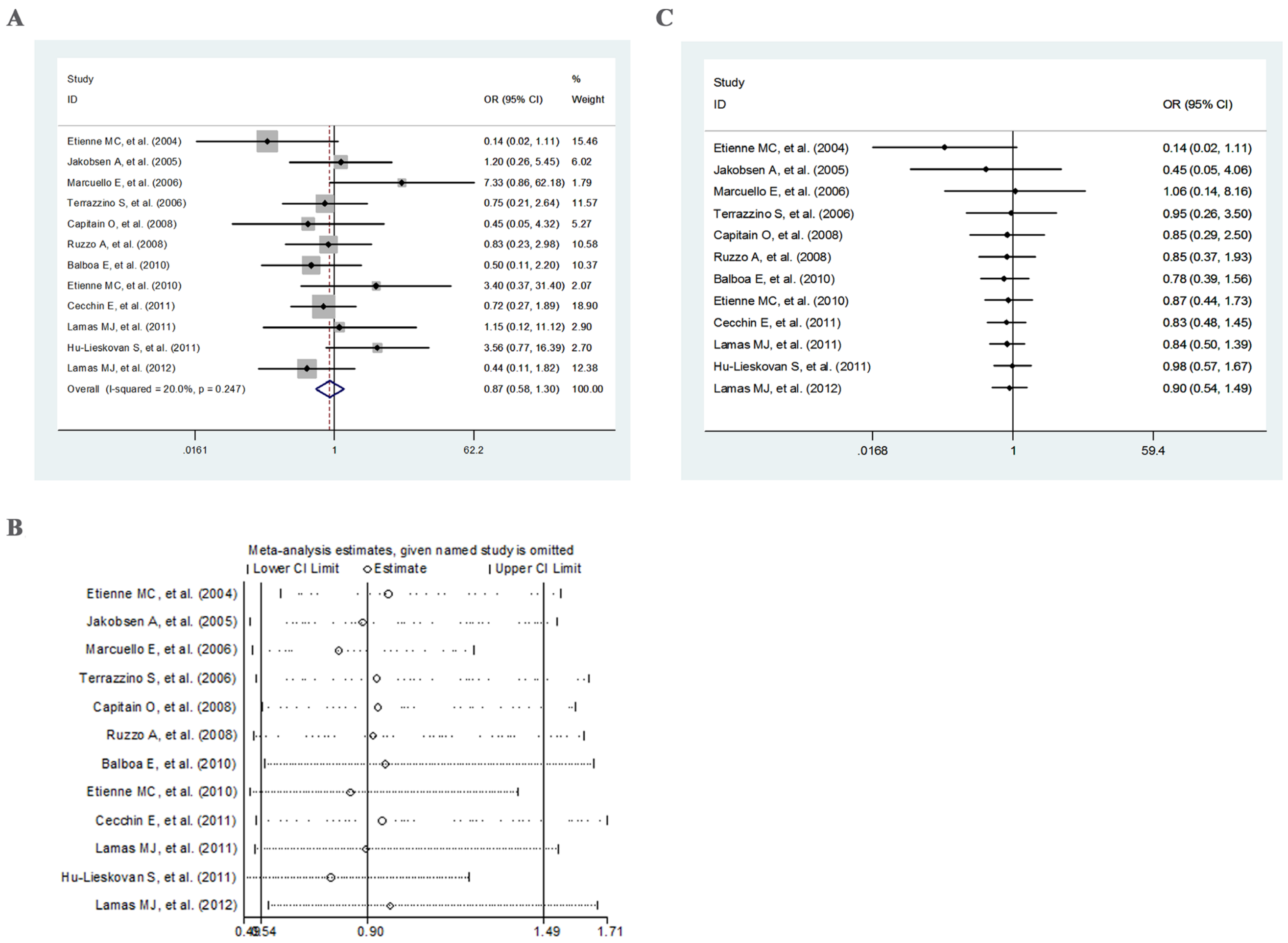

Supplementary Figure 4: Forest plot (**A**), sensitivity analysis (**B**) and cumulative meta-analysis (**C**) for the recessive model of *MTHFR* A1298C polymorphism and response to fluoropyrimidine-based chemotherapy.
